# Treatment Duration for Patients with Drug-Resistant Tuberculosis, United States

**DOI:** 10.3201/eid1807.120261

**Published:** 2012-07

**Authors:** Carla A. Winston, Kiren Mitruka

**Affiliations:** Centers for Disease Control and Prevention, Atlanta, Georgia, USA

**Keywords:** drug resistance, tuberculosis, TB, treatment duration, antimicrobial drug treatment, antimicrobial resistance, United States, Mycobacterium tuberculosis, monoresistant TB strains, isoniazid, rifampin, multidrug-resistant TB, MDR TB, tuberculosis and other mycobacteria

**To the Editor:** In the United States, almost 80% of tuberculosis (TB) cases are diagnosed on the basis of positive culture results for *Mycobacterium tuberculosis*, and >90% of initial isolates are tested for drug susceptibilities (*1**,**2*). Recommended treatment durations are 6–9 months for patients with isoniazid- and rifampin-susceptible TB; <18 months for patients with rifampin-monoresistant TB; and, following culture conversion, 18–24 months for patients with isoniazid- and rifampin-resistant TB (*3*). Appropriately completed TB treatment maximizes patient and public health benefits and minimizes adverse events and costs (*3*). We examined treatment duration by drug resistance pattern among a national cohort of case-patients with TB diagnosed in the United States.

We analyzed routinely collected data from the Centers for Disease Control and Prevention’s National TB Surveillance System. To ensure that all patients had at least 3 years of follow-up, we examined cases of culture-positive TB verified in 2006. We calculated treatment duration for patients who were alive and had initiated TB therapy at diagnosis and who had results for initial drug susceptibility testing. Treatment duration was calculated by subtracting the therapy start date from the therapy end date. The 15th day of the month was assigned as the day treatment started or ended if that information was missing. Patients who did not complete therapy were censored as of the last known follow-up. We categorized cases as isoniazid monoresistant; rifampin monoresistant; multidrug resistant (MDR), defined as resistant to at least isoniazid and rifampin; or drug susceptible, defined as susceptible to isoniazid, rifampin, and ethambutol and with no known resistance to pyrazinamide (i.e., pyrazinamide susceptible or missing test results). Survival distributions by drug-resistance pattern were estimated by using Kaplan-Meier analysis and compared by using log-rank test statistics. Patient characteristics were compared by using χ^2^ tests or, when cell sizes were <5, Fisher exact tests.

Of 13,734 TB cases reported in 2006, 10,747 (78.3%) were confirmed by culture. Of the 10,747 patients with culture-confirmed TB, 10,120 (94.2%) were alive and had initiated TB therapy at diagnosis and had start and end therapy dates and initial drug susceptibility results. Duration of treatment was calculated for 9,734 (96.2%) cases, of which, 8,973 (92.2%) were classified as drug-susceptible, 618 (6.3%) as isoniazid-monoresistant, 24 (0.2%) as rifampin-monoresistant, and 119 (1.2%) as MDR TB. The remaining 386 (3.8%) cases were excluded from analysis because the patients had pyrazinamide-monoresistant TB, suggestive of *Mycobacterium bovis* infection (165), or they were missing susceptibility testing results for isoniazid, rifampin, or ethambutol (112) or had other resistance patterns (109).

At 12 months, the cumulative completion of therapy among patients with drug-susceptible, isoniazid-monoresistant, rifampin-monoresistant, or MDR TB was 87.6%, 81.0%, 17.4%, and 1.9%, respectively (Figure). At 24 months, 73.9% of patients with rifampin-monoresistant TB and 40.2% with MDR TB had completed treatment. Treatment duration was shortest for patients with drug-susceptible TB (median 252 days), compared with a median of 274, 555, and 766 days for patients with isoniazid-monoresistant, rifampin-monoresistant, and MDR TB, respectively. Differences in treatment duration based on drug susceptibility were significant (p<0.001) for all comparisons. The MDR TB group included 4 extensively drug-resistant cases (also resistant to any fluoroquinolone and >1 of the injectable drugs capreomycin, kanamycin, or amikacin) (*4*); no remarkable change in duration of treatment resulted when those 4 cases were removed from analysis.

**Figure:**
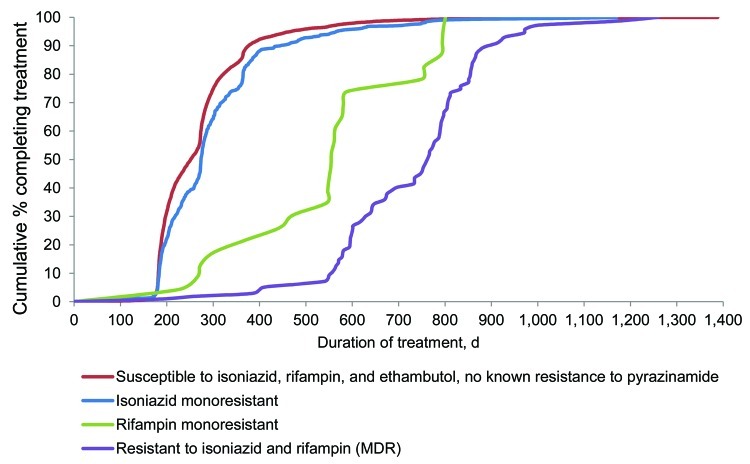
Treatment duration, by drug-resistance pattern, among reported tuberculosis case-patients who completed treatment, United States, 2006. Cases were among patients who were alive and initiated therapy at diagnosis and who had start and end therapy dates as well as results for initial drug susceptibility testing to isoniazid, rifampin, and ethambutol. Susceptibility testing was conducted on culture-positive *Mycobacterium tuberculosis* isolates from any specimen type.

The surveillance system captures only the initial treatment regimen; thus, we could not assess changes to treatment regimens in response to drug susceptibility test results or treatment nonadherence. We observed no difference in history of prior TB; HIV infection; or miliary, meningeal, pediatric, or bone and joint TB among case-patients with isoniazid-resistant versus drug-susceptible TB (p>0.12 for all comparisons). TB treatment recommendations in the United States emphasize completion within 12 months of initiating therapy, with exceptions for rifampin-resistant TB, meningeal TB, and disseminated disease in pediatric patients (children <15 years of age) (*1**,**5*). We found no change in treatment duration by drug-resistance pattern after removing cases of meningeal TB or cases in children from analysis.

The length of TB treatment duration in the United States has improved since therapy outcomes were first recorded in the National TB Surveillance System in 1993. In our study, 90% of case-patients with drug-susceptible TB completed therapy within 373 days, compared with 671 days in 1993 (*6*), and 90% of patients with isoniazid-monoresistant TB completed therapy within 432 days. Although the percentage of MDR TB cases in the United States has declined since 1993, drug resistance remains a serious concern because the percentage of isoniazid-monoresistant TB cases has remained stable (*7*). Our analysis suggests that despite the effectiveness of rifampin-containing regimens and an apparent lack of clinical differences to justify extending therapy, longer treatment durations persist among patients with isoniazid-monoresistant TB (*8*). In our cohort study, <75% of patients with rifampin-monoresistant TB and 40% with MDR TB completed therapy within 24 months, suggesting no improvement since 1993 in the length of treatment duration for rifampin-resistant TB strains (*6*).

## References

[1] Centers for Disease Control and Prevention. Reported tuberculosis in the United States, 2008. Atlanta (GA): US Department of Health and Human Services; 2009.

[2] LoBue PA, Enarson DA, Thoen TC. Tuberculosis in humans and its epidemiology, diagnosis and treatment in the United States. Int J Tuberc Lung Dis. 2010;14:1226–32. 20843412

[3] Blumberg HM, Burman WJ, Chaisson RE, Daley CL, Etkind SC, Friedman LN, American Thoracic Society/Centers for Disease Control and Prevention/Infectious Diseases Society of America: treatment of tuberculosis. Am J Respir Crit Care Med. 2003;167:603–62. 10.1164/rccm.167.4.603 12588714

[4] Centers for Disease Control and Prevention. Notice to readers: revised definition of extensively drug-resistant tuberculosis. MMWR Morb Mortal Wkly Rep. 2006;55:1176.

[5] Mitruka K, Winston CA, Navin TR. Predictors of failure in timely tuberculosis treatment completion, United States. Int J Tuberc Lung Dis. 2012;16. In press. 10.5588/ijtld.11.0814 22668774

[6] Bloch AB, Cauthen GM, Simone PM, Kelly GD, Dansbury KG, Castro KG. Completion of tuberculosis therapy for patients reported in the United States in 1993. Int J Tuberc Lung Dis. 1999;3:273–80. 10206496

[7] Hoopes AJ, Kammerer JS, Harrington TA, Ijaz K, Armstrong LR. Isoniazid-monoresistant tuberculosis in the United States, 1993 to 2003. Arch Intern Med. 2008;168:1984–92. 10.1001/archinte.168.18.1984 18852399

[8] Cattamanchi A, Dantes RB, Metcalfe JZ, Jarlsberg LG, Grinsdale J, Kawamura LM, Clinical Characteristics and treatment outcomes of patients with isoniazid-monoresistant tuberculosis. Clin Infect Dis. 2009;48:179–85. 10.1086/595689 19086909 PMC2756509

